# Editorial for the Special Issue “Detoxification Mechanisms in Insects”

**DOI:** 10.3390/toxics11080691

**Published:** 2023-08-10

**Authors:** Ahmed A. A. Aioub, Mohamed-Bassem Ali Ashour

**Affiliations:** Plant Protection Department, Faculty of Agriculture, Zagazig University, Zagazig 44511, Egypt

Insects are faced with numerous toxins (xenobiotics) as they go through life, some produced naturally by plants (sometimes called allelochemicals) and some produced by humans (insecticides). To survive these natural toxins, various detoxification mechanisms have evolved in insects. These same mechanisms also sometimes allow insects to overcome insecticides, and the level and type of mechanisms differ greatly. This results in differing toxicity among different stages, populations, and species of insects. Knowledge of detoxification allows us to better incorporate chemical resistance mechanisms in crops and to better select insecticides that will be effective when applied. Detoxification can be divided into three phases: phase I, phase II (involving metabolizing enzymes), and phase III (involving transporters). Cytochrome P450 monooxygenase (Cyp 450), glutathione S-transferase (GST), and carboxylesterase (CarE) are the primary enzymes involved in phase I and phase II detoxification processes, whereas phase III is dominated by ATP-binding cassette (ABC) transporters. Phase I reactions consist of oxidation, hydrolysis, and reduction.

In the present work, five papers published in this Special Issue are summarized, providing a picture of the role of detoxification mechanisms in insects. Two studies assessed the combined effects of different insecticides on the detoxification enzymes of *Spodoptera littoralis*. Moustafa et al. [1] showed six insecticides (chlorpyrifos, methomyl, alpha-cypermethrin, hexaflumeron, *Bacillus thuringiensis*, and spinosad) had significantly different activity levels on the determination of CarE, Cyp 450, and GST for three field strains of *S. littoralis* compared with the susceptible strain. Moreover, El-Sayed et al. [2] explored how combining enzyme inhibitors triphenyl phosphate (TPP), diethyl maleate (DEM), and piperonyl butoxide (PBO) with chemical insecticides may be beneficial in overcoming the mechanisms of resistance in insects. The combined action of TPP, DEM, and PBO with cypermethrin and spinosad exhibited synergistic action versus the fourth larval instars of *S. littoralis* and inhibition of CarE, GST, and Cyp 450.

Another study by Moustafa et al. [3] was initiated to evaluate the larvicide activity and biochemical of chlorantraniliprole (CHP) and indoxacarb against *Mamestra brassicae*. The results revealed that both chlorantraniliprole and indoxacarb have insecticidal activities, with LC_10_, LC_30_, and LC_50_ values against *M. brassicae* of 0.001, 0.03, 0.35 mg/L, and 0.08, 0.50, 1.71 mg/L, respectively. Additionally, sublethal doses of the above insecticides significantly reduced ß-esterase and GST activities but had no significant effect on Cyp 450.

The next paper included in this Special Issue evaluated the digestive and anti-toxicological physiological impacts of *Justicia adathoda*, *Pongamia glabra*, *Annona squamosa*, and *Ipomea carnea* crude extracts and their AgNPs (0, 1.25, 2.5, 5.0, and 10%) and the commercial botanical pesticide vijayneem (0.03%) in *Phenacoccus solenopsis* [4]. The results show that the total body of *P. solenopsis* contains trypsin, pepsin, invertase, lipase, and amylase, and *J. adathoda* and *I. carnea* aqueous extracts considerably decreased the protease and phospholipase A2 levels and *A. squamosa* aqueous extract dramatically increased the trehalase level in a dose-dependent manner. The enzyme levels were dramatically decreased by *P. glabura* AgNPs (invertase, protease, trehalase, lipase, and phospholipase A2); *I. carnea* AgNPs (invertase, lipase, and phospholipase A2); *A. squamosa* AgNPs (protease, phospholipase A2); and *J. adathoda* AgNPs (protease, lipase, and acid phosphatase). Plant extracts and their AgNPs significantly reduced *P. solenopsis* esterase and lactate dehydrogenase levels in a dose-dependent manner. 

Finally, this Special Issue also includes a study by Aioub et al. [5] that identified and characterized 31 GST genes (SfGSTs) and determined the expressions of 28 out of these 31 GST genes by qRT-PCR under *S. frugiperda* under emamectin benzoate (EBZ) and chlorantraniliprole (CHP) stress. The results displayed that the LC_50_ values of EBZ and CHP were 0.029 and 1.250 mg/L, respectively, after 24 h of exposure. Moreover, SfGSTe10 and SfGSTe13 stood out, with the highest expression after EBZ and CHP treatments. Furthermore, the molecular docking study showed EBZ and CHP have a high binding affinity with SfGSTe10, with docking energy values of −24.41 and −26.72 kcal/mol, respectively, and SfGSTe13, with docking energy values of −26.85 and −26.78 kcal/mol, respectively.

In summary, the five manuscripts in the present Special Issue demonstrate small steps towards our continued understanding of the effects of insecticides on detoxification enzymes in insects. This area of research is far from complete, and greater efforts are needed from the scientific community to complete the full picture of the mechanisms of insecticide inside insects.

We would like to express our appreciation to all authors for contributing their unique work to this Special Issue, as well as the reviewers, who played a crucial role in determining the relevance of the submissions and enhancing their quality. Moreover, we cannot forget to thank the *Toxics* editors for their gracious invitation and, in particular, Susie Deng of the *Toxics* Editorial Office for her priceless and devoted support.
